# Self-protective and self-sacrificing preferences of pedestrians and passengers in moral dilemmas involving autonomous vehicles

**DOI:** 10.1371/journal.pone.0261673

**Published:** 2021-12-23

**Authors:** Maike M. Mayer, Raoul Bell, Axel Buchner

**Affiliations:** Department of Experimental Psychology, Heinrich Heine University Düsseldorf, Düsseldorf, Germany; Tsinghua University, CHINA

## Abstract

Upon the introduction of autonomous vehicles into daily traffic, it becomes increasingly likely that autonomous vehicles become involved in accident scenarios in which decisions have to be made about how to distribute harm among involved parties. In four experiments, participants made moral decisions from the perspective of a passenger, a pedestrian, or an observer. The results show that the preferred action of an autonomous vehicle strongly depends on perspective. Participants’ judgments reflect self-protective tendencies even when utilitarian motives clearly favor one of the available options. However, with an increasing number of lives at stake, utilitarian preferences increased. In a fifth experiment, we tested whether these results were tainted by social desirability but this was not the case. Overall, the results confirm that strong differences exist among passengers, pedestrians, and observers about the preferred course of action in critical incidents. It is therefore important that the actions of autonomous vehicles are not only oriented towards the needs of their passengers, but also take the interests of other road users into account. Even though utilitarian motives cannot fully reconcile the conflicting interests of passengers and pedestrians, there seem to be some moral preferences that a majority of the participants agree upon regardless of their perspective, including the utilitarian preference to save several other lives over one’s own.

## Introduction

As autonomous driving technologies constantly improve, the introduction of automated and eventually fully autonomous vehicles into daily traffic for private and commercial uses is in the progress of being realized [1]. Many governments around the world are aware of the economic importance of automated driving and support the development and introduction of autonomous driving technologies [cf. 2–4]. The expected improvements to safety, accessibility of transportation, and traffic flow [cf. 1, 5] spur the interest in these technologies. Given the high number of annual traffic fatalities [globally about 1,35 million in 2016, 6, United States: 36,560 in 2018, 7, European Union: about 22,800 [estimated] in 2019, 8] and human error as a major cause of accidents [9], the prospect of increased traffic safety [e.g., 1, 5] is one of the most salient advantages of automated driving [e.g., 10]. Nonetheless, autonomous vehicles cannot avoid all accidents—regardless of the system’s reliability or the frequency of such incidents—as they share the roads with other road users such as pedestrians, human drivers, and animals whose behaviors are difficult to predict [11–14]. Thus, in order to enable autonomous vehicles to participate in public traffic, it is necessary to program them for how to handle accidents [12, 14]. While human drivers have to make split-second decisions in critical traffic situations, autonomous vehicles provide the unique opportunity for considering in advance how critical situations should be handled [10, 15–18]. However, there are two sides to every coin. Designing an algorithm to handle accidents and implementing it in numerous vehicles implies the danger that any intended or unintended bias introduced to the system may determine decisions about life and death [10, 19]. This is a particularly delicate matter as autonomous vehicles may face difficult moral decisions [12, 13, 15, 20] such as whom to harm or even sacrifice in an inevitable accident.

Various partially conflicting norms and principles—among which deontology and utilitarianism are probably the most prominent—affect moral decision making [e.g., 21, 22] including decisions about how autonomous vehicles should handle unavoidable collisions comprising moral aspects [e.g., 11, 14, 23–25]. While deontology focuses on moral rules [such as obligations and prohibitions, e.g., 26], utilitarianism is concerned with the outcome of particular actions. Following a deontological approach, an action is morally acceptable and permissible if it is consistent with moral norms (e.g., “You shall not kill”) whereas from a utilitarian point-of-view an action is permissible and acceptable if it maximizes utility [e.g., 27, 28] by minimizing negative consequences such as overall damage or harm.

A popular approach to studying moral decision making is the Trolley Problem [29–31]. In the original version of this moral dilemma, a runaway trolley is speeding down the tracks. On the tracks, there are five people who are unable to move out of the way in time. It is, however, possible to lead the trolley to a side track where it will kill only one person. Is it morally acceptable to kill this person to save five other lives? It is easy to envision similar scenarios with autonomous vehicles: Imagine that five pedestrians suddenly step into a road upon which an autonomous vehicle is driving. The autonomous vehicle cannot come to a stop in time; it only has the option to either crash into the group of pedestrians or swerve to the side into an obstacle, killing the passenger. Should the autonomous vehicle sacrifice the passenger to save the lives of the pedestrians or should it sacrifice the pedestrians, leaving the passenger unharmed?

The question of how to program autonomous vehicles for handling accidents that require moral decisions has sparked interdisciplinary research and considerable debate. Scenarios modeled after the Trolley Problem have become standard tools to investigate moral dilemmas involving autonomous vehicles [e.g., 10, 14, 32]. Most prominently, in the Moral Machine experiment [13] different scenarios were tested against each other, involving millions of people from more than 200 countries. Among the strongest moral preferences identified in this study was the utilitarian preference to spare more lives, but there was considerable variation in preferences. Scenarios modeled after the Trolley Problem cannot serve as a blueprint for how to program autonomous vehicles [e.g., 32, 33], but they can serve to identify morally relevant properties of accident scenarios [e.g., 34], to test ethical theories [e.g., 16], and to examine moral intuitions and moral decision making [e.g., 32–34]. This is particularly relevant as public acceptance is a prerequisite for the success of autonomous vehicles [12, 13, 17, 18, 25, 32, 34, 35]. The programming of autonomous vehicles for handling moral decisions in accident scenarios requires careful consideration of what decisions people are willing to accept.

While from a societal perspective it may seem desirable that the actions of autonomous vehicles are guided by moral norms and aim at saving a maximum number of lives, research suggests that people’s preferences are not only guided by moral and utilitarian considerations but also by self-protective tendencies. From an evolutionary perspective, it seems possible that self-protective tendencies are ingrained in cognitive decision making [36] which implies that people show a preference for actions that protect their own life. In line with this prediction, Bonnefon et al.’s [35] participants indicated that they were unwilling to buy utilitarian autonomous vehicles for themselves despite agreeing that utilitarian programming represents a good—or morally superior—approach. Similarly, Liu and Liu [37] observed that participants showed a higher intention to use autonomous vehicles programmed to protect their passengers and were overall more willing to pay extra money for this type of self-driving technology compared to utilitarian autonomous vehicles. This pattern of results suggests that determining the actions of autonomous vehicles in critical accidents may represent a social dilemma [e.g., 18, 35, 38]. While it may be desirable for society to minimize the number of people harmed in accidents, customers might display a selfish interest to protect their own lives. In consequence, automated vehicles that value the lives of the passengers higher than that of other road users may prevail in the market. However, there are also results suggesting that there may be a limit to people’s selfishness. Faulhaber et al. [18] observed an increasing willingness to self-sacrifice with an increasing number of potential victims that could be saved by a self-sacrifice. Taken together, people’s preferences may be characterized as utilitarianism biased by self-protective tendencies. Specifically, most people agree that an autonomous vehicle should sacrifice their own life if this action saves the lives of many other people, but this utilitarian preference to reduce harm and save lives is limited by a tendency to value one’s own physical safety more than that of another person. In consequence, a number of other people’s lives have to be at stake before self-sacrifice is considered the preferred option.

So far, many governments have refrained from touching upon the moral dilemmas that may arise from unavoidable accidents involving the deaths of passengers and other road users while they have realized the importance of autonomous driving and have discussed concerns of traffic safety [2–4]. A notable exception is the German Federal Ministry of Transport and Digital Infrastructure whose Ethics Commission has published official guidelines for how autonomous vehicles should be programmed to handle morally relevant situations [39]. This is all the more interesting as these guidelines do not always align with the laypeople’s preferences found in experimental studies. For example, the guidelines neither prescribe nor prohibit sacrificing few to protect many although several studies demonstrate that participants tolerate or even prefer a utilitarian approach for autonomous vehicles [e.g., 13, 18, 25, 35, 40, 41]. The guidelines also state that parties who do not generate a mobility risk (e.g., pedestrians) must not be sacrificed to save those generating that risk (the passengers of the autonomous vehicles). This suggestion is especially noticeable because it explicitly distinguishes between the safety concerns of different road users. However, most research has focused only on the perspectives of passengers and observers [e.g., 13, 18, 35, 40]. This is a narrowed perspective as other road users are also directly affected by the actions of autonomous vehicles and may well differ in their preferences for certain outcomes of moral dilemmas from passengers of autonomous vehicles. The perspective of the pedestrian seems particularly important because pedestrians represent the largest group of non-motorized road users [42].

To date, there are only few studies investigating to what degree moral preferences of non-motorized road users differ from those of passengers regarding the programming of autonomous vehicles. In the study of Kallioinen et al. [43], participants experienced the perspectives of passengers and pedestrians from the first-person perspective in an immersive virtual environment. The results lent support to the hypothesis that pedestrians have a self-protective preference for the passenger to be sacrificed. The study also hints at the possibility that there are moral principles that transcend these self-protective biases as both passengers and pedestrians agreed upon the utilitarian principle that the option that preserves most lives is to be preferred. However, when interpreting these findings it is important to consider that Kallioinen et al. [43] tested the influence of perspective in an immersive environment in which the pedestrians saw the approaching car from the first-person perspective. It is thus possible to speculate that the saliency of the imminent threat for survival may have amplified self-protective tendencies in this study.

Relevant decisions about purchasing a car or about determining algorithms for dealing with accidents are often made in the absence of imminent threat. It is thus interesting to test whether the same effects can be found when people reason about abstract scenarios in which the threat to survival is less salient. Here it is relevant that Frank et al. [44] cued participants into the perspective of passengers, pedestrians, and observers when judging abstract scenarios of moral dilemma situations with autonomous vehicles. They observed self-protective biases in the sense that participants who were cued into the perspective of the passenger were more willing to sacrifice the pedestrian than participants who were cued into the perspective of the pedestrian. However, these self-protective tendencies were less pronounced than one might think. First, the majority of the participants favored sacrificing the passenger to save the pedestrian even when evaluating the scenarios from the passenger perspective, which suggests that there are limitations to the degree to which moral judgments are biased by self-protective tendencies. When the numbers of passengers and pedestrians were manipulated, the participants expressed preferences in line with the utilitarian principle that it is preferable to sacrifice one life to save many others.

Here, we revisited this issue by testing, across four experiments (Experiments 1a to 2b), people’s decisions in moral dilemmas with autonomous vehicles in which people’s self-protective tendencies are put against the utilitarian preference of saving the maximum number of lives. This was done by systematically manipulating the number of pedestrians on the road (Experiments 1a and 1b) and the number of passengers inside the autonomous vehicle (Experiments 2a and 2b). In each experiment, participants were randomly assigned to one of three perspectives (passenger, pedestrian, observer) and asked to indicate their preferred course of action for different accident scenarios with autonomous vehicles. To anticipate, we observed a strong and robust influence of perspective on the preferred action of autonomous vehicles in moral dilemma situations. However, even though differences among perspectives persisted, self-sacrificing tendencies dominated over self-protective tendencies when many lives could be saved by a self-sacrifice. In Experiment 3, we tested whether these self-sacrificing preferences are due to a social desirability bias by employing an indirect questioning technique [45]. The hypothesis that people’s self-sacrificing preferences are due to a social desirability bias had to be rejected, which supports the validity of people’s stated preference to self-sacrifice when the utilitarian principle strongly favors this option.

## Experiment 1a

### Method

The experiment was conducted online. It was programmed with *SoSci Survey* [46] and was made available for participation at www.soscisurvey.de. Completing the experiment took about 15 minutes. This experiment and its subsequent replications were approved by the ethics committee of the Faculty of Mathematics and Natural Sciences at Heinrich Heine University Düsseldorf and all reported studies were conducted in accordance with the Declaration of Helsinki and its later amendments. Written informed consent was obtained from all participants prior to participation in each study.

#### Participants

Participants were recruited on campus at Heinrich Heine University Düsseldorf and via online advertisements. As a compensation for participating, all participants could enter a lottery to win one of three € 20 gift cards for a popular online store. Psychology students received course credit for participation. Of the participants who started the study, 62 did not complete the experiment, four were not of legal age (a requirement for being able to consent to the processing of one’s data in Germany), and 26 did not respond to all items. The final sample included the data of 325 participants (248 female, 76 male, one diverse) aged between 18 and 61 years (*M* = 24, *SD* = 7). A sensitivity analysis performed with G*Power [47] showed that, with a total sample size of *N* = 325 participants and 15 observations per participant in the experiment, small effects of size *w* = .06 [48] could be detected at an α level of .05 with a statistical power of 1—β = .95 in the model-based statistical tests (see Results section) for the overall comparison among perspectives (*df* = 4). Participants were randomly assigned to one of three perspectives—pedestrian (*n* = 109), observer (*n* = 111), or passenger (*n* = 105)—from which they were asked to evaluate the moral dilemma scenarios. More detailed information about the sample—including information about the participants’ trait empathy [German version of the Interpersonal Reactivity Index; 49], affinity for technology [usage of, and opinion on, electronic devices; TA-EG; 50], and acceptance of autonomous vehicles [based on the questionnaire of 51, 52]—are available at the Open Science Framework (OSF) project page (https://osf.io/4xhz7/).

#### Material and procedure

**Fig 1:**
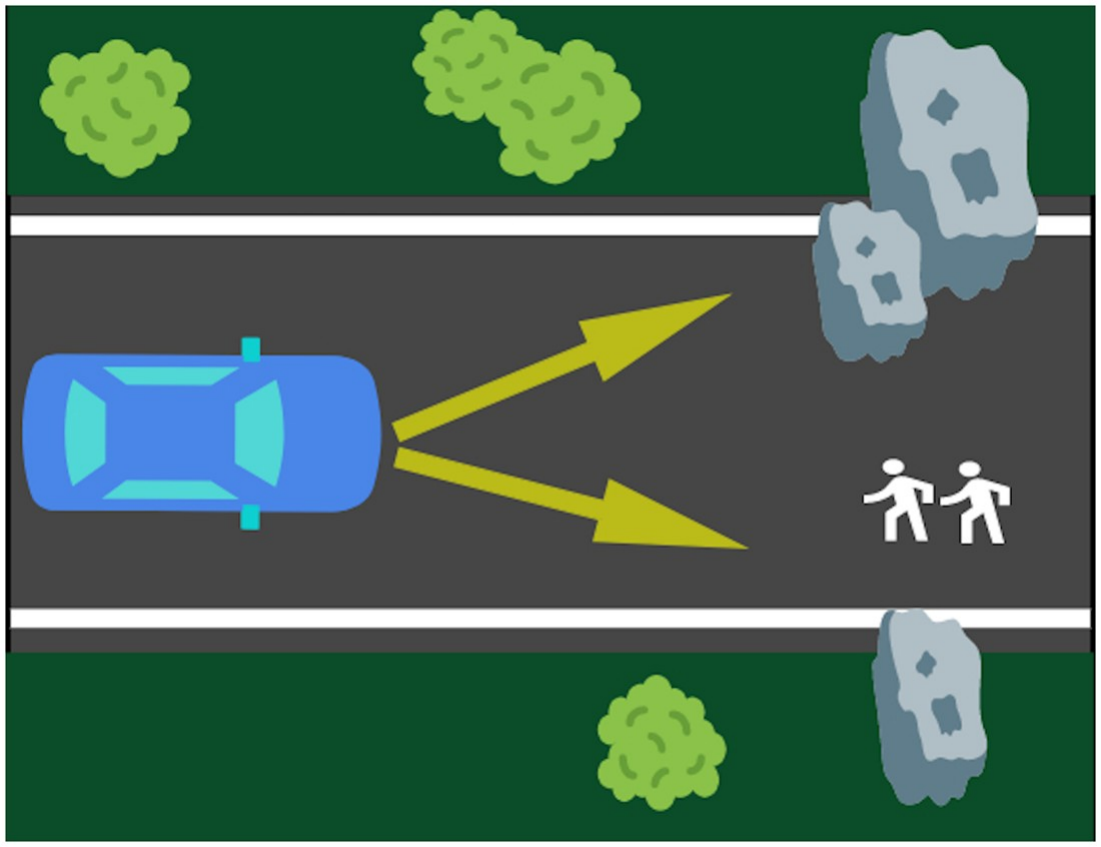
Example of an illustration of an accident scenario. In this example, the passenger-to-pedestrian ratio is 1:2, which means that the life of one passenger is weighed against that of two pedestrians. The visual illustrations of the scenarios were created using Microsoft PowerPoint.

Participants were first provided with a definition of autonomous vehicles. Autonomous vehicles were defined as self-driving cars capable of participating in traffic on their own without the need of human intervention or back-up. Furthermore, participants were asked to adopt the perspective of a pedestrian, an observer, or a passenger (between-subjects factor). Two example dilemmas were described in detail. In the experiment proper, different moral dilemma scenarios were presented in random order, one at a time. Each scenario comprised an autonomous vehicle driving down a single-lane road with one or more pedestrians and an obstacle (such as a boulder) on the road ahead (see Fig 1 for an example). Participants were instructed that the vehicle could not come to a stop in time and an accident was inevitable. Only two options remained: The autonomous vehicle would collide either with the obstacle—killing the passenger—or with the pedestrian/s—killing them in the process. The scenarios were depicted as abstract sketches from a bird’s eye view and showed the vehicle as well as the pedestrians and obstacles in its path. The two options available to the autonomous vehicle were illustrated with arrows. In each scenario either one, two, five, or ten pedestrians were on the road. The different numbers of pedestrians were presented in four different environments, yielding 16 different scenarios in total. The position of the vehicle (right or left side of the image) and of the pedestrians (upper or lower half of the road) was counterbalanced for each combination of number of pedestrians and environment. The experiment thus employed a 3 (perspective: pedestrian, observer, passenger; between-subjects factor) × 4 (passenger-to-pedestrian ratio: 1:1, 1:2, 1:5, 1:10; within-subjects factor) design.

Immediately below the image of the scenario, a short reminder of the respective perspective was given (“You are the/a pedestrian/observer/passenger.”). Then, participants were asked: “How should the autonomous vehicle act in your opinion?” Participants had to choose whether it should “sacrifice the pedestrian/s” or “sacrifice the passenger”.

The scenario and the question were presented for a maximum of 15 seconds. If participants failed to answer the question in that time span, the next scenario was automatically presented. Data sets of participants failing to evaluate all scenarios were marked as incomplete and were excluded from analysis.

### Results

**Fig 2:**
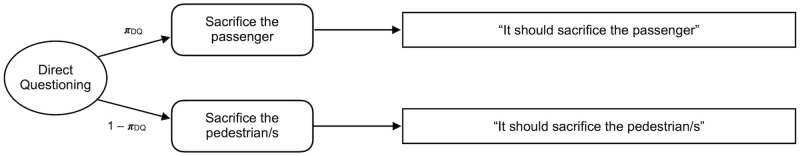
The multinomial processing tree model used in Experiments 1a and 1b. The rectangles on the right represent the answer categories available in each condition. Parameter π_DQ_ represents the parameter estimate for the preference that the autonomous vehicle should sacrifice the passenger instead of the pedestrian/s. Separate model trees were necessary for each combination of the 3 (perspective: pedestrian, observer, passenger) × 4 (passenger-to-pedestrian ratio: 1:1, 1:2, 1:5, 1:10) design. Note that the model corresponds to the model representing the direct questioning approach in Experiment 3.

**Fig 3:**
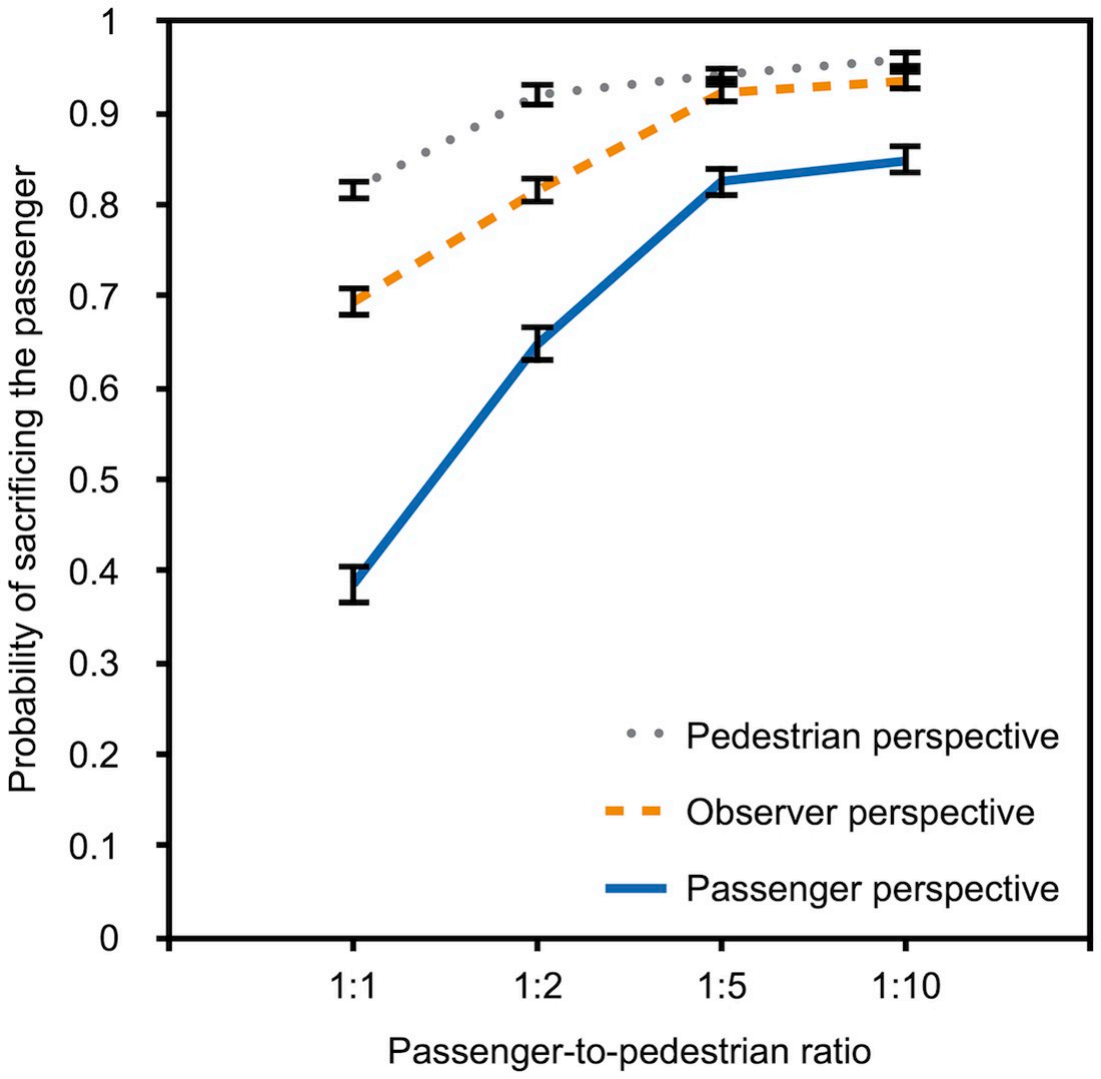
Descriptive data for Experiment 1a. The probability of sacrificing the passenger rather than the pedestrian/s is depicted as a function of passenger-to-pedestrian ratio (1:1, 1:2, 1:5, and 1:10) and perspective (pedestrian, observer, and passenger). The error bars represent bootstrapped standard errors.

We used *multiTree* [53] to estimate the preferences for sacrificing the passenger for each passenger-to-pedestrian ratio and each perspective based on the observed answer frequencies. To maintain consistency in the analysis with Experiment 3, we used the simple model depicted in Fig 2 to estimate the participants’ preference—in terms of a probability between 0 and 1—to sacrifice the passenger as a function of the perspective (pedestrian, observer, passenger) and the passenger-to-pedestrian ratio (1:1, 1:2, 1:5, 1:10). Participants’ preferences are shown in Fig 3. Due to technical difficulties with the display of one scenario, three (instead of four) responses were analyzed for the passenger-to-pedestrian ratio of 1:5.

Fig 3 suggests that the preference for sacrificing the passenger increases with an increasing number of pedestrians that can be saved by this action. The results also suggest that the preference to sacrifice the passenger differs as a function of perspective. Participants who had adopted the perspective of a pedestrian showed the strongest preference for sacrificing the passenger while participants who had adopted the perspective of a passenger showed the lowest preference for sacrificing the passenger at all levels of the passenger-to-pedestrian-ratio variable. We used *multiTree* [53] to compare the preferences among conditions. The α level for these analyses was set to .05 and Bonferroni-Holm adjusted [54]. Confirming the visual impression from Fig 3, the multinomial analysis confirmed that the preferences of pedestrians differed significantly from those of the passengers, *G*^2^(4) = 326.60, *p* < .001, *w* = .26. The preferences of pedestrians, *G*^2^(4) = 43.01, *p* < .001, *w* = .09, and passengers, *G*^2^(4) = 146.58, *p* < .001, *w* = .17, differed from those of observers.

Next, we compared the preferences for sacrificing the passenger among the three perspectives at each level of the passenger-to-pedestrian-ratio variable. Again, the α level for these analyses was set to .05 and Bonferroni-Holm adjusted [54]. The test statistics are reported in Table 1. All pairwise comparisons are significant with the exception of the comparisons between pedestrians and observers at the passenger-to-pedestrian ratios of 1:5 and 1:10, which is probably simply due to the fact that an overwhelming majority of the pedestrians and observers preferred the utilitarian option of sacrificing the passenger in order to save the lives of five or more pedestrians.

**Table 1:** Comparisons among perspectives separately for each passenger-to-pedestrian ratio in Experiment 1a.

	1:1	1:2	1:5	1:10
Pedestrian vs. passenger	*G*^2^(1) = 172.80	*G*^2^(1) = 99.65	*G*^2^(1) = 22.12	*G*^2^(1) = 32.04
	*p* < .001*	*p* < .001*	*p* < .001*	*p* < .001*
	*w* = .19	*w* = .14	*w* = .07	*w* = .08
Pedestrian vs. observer	*G*^2^(1) = 18.09	*G*^2^(1) = 21.35	*G*^2^(1) = 1.04	*G*^2^(1) = 2.54
	*p* < .001*	*p* < .001*	*p* = .308	*p* = .111
	*w* = .06	*w* = .07	*w* = .01	*w* = .02
Observer vs. passenger	*G*^2^(1) = 83.85	*G*^2^(1) = 31.36	*G*^2^(1) = 14.02	*G*^2^(1) = 17.35
	*p* < .001*	*p* < .001*	*p* < .001*	*p* < .001*
	*w* = .13	*w* = .08	*w* = .05	*w* = .06

The α level was set to .05 and Bonferroni-Holm adjusted [54]. Significant comparisons are indicated by an asterisk.

### Discussion

Experiment 1a confirms that preferences about the actions of autonomous vehicles in moral dilemmas strongly depend on perspective. Participants who evaluated the scenarios from the perspective of the pedestrian consistently displayed the highest preference for sacrificing the passenger while participants who were cued into the perspective of the passenger displayed the lowest preference for sacrificing the passenger, confirming the existence of self-protective tendencies in both pedestrians and passengers. With an increasing number of pedestrians who could be saved by sacrificing the passenger, the preference for sacrificing the passenger increased in all groups, suggesting a utilitarian preference for sacrificing one life to save several others. However, it seems noticeable that differences among the perspectives were not completely eliminated even at the most extreme passenger-to-pedestrian ratios (with the exception of pedestrians and observers who agreed that five and more pedestrians should be saved at the sacrifice of one passenger), suggesting that the utilitarian preference for saving a maximum number of lives does not completely eliminate the self-protective bias.

Given the current discussion about the robustness of psychological findings [55], we deemed it necessary to replicate the findings before drawing firm conclusions. To test the robustness of the findings, Experiment 1b served as a close replication of Experiment 1a, with the main difference to Experiment 1a being that participants were recruited from an online research panel.

## Experiment 1b

### Method

#### Participants

Participants were recruited from the online research panel of respondi AG based in Cologne, Germany. Participants received a small monetary compensation for participating in the study. Of the participants who started the study, 30 did not complete the experiment, four indicated that they had insufficient German language skills or were unable to properly read the text on the screen, 42 did not respond to all items, and five were excluded because they gave identical answers to all items of the three questionnaires at the end of the study and thus seemed to have “clicked through” the experiment. The final sample included the data of 365 participants (172 female, 193 male) aged between 18 and 69 years (*M* = 49, *SD* = 14). With this sample size, effects of size *w* = .06 could be detected at an α level of .05 with a statistical power of 1—β = .95 in the overall comparison among perspectives (*df* = 4). As in Experiment 1a, participants were randomly assigned to one of three perspectives—pedestrian (*n* = 118), observer (*n* = 124), or passenger (*n* = 123)—from which they were asked to evaluate the moral dilemma scenarios. Additional information about the sample is reported at the OSF project page (https://osf.io/4xhz7/).

#### Material and procedure

Material and procedure were identical to those of Experiment 1a.

### Results

**Fig 4:**
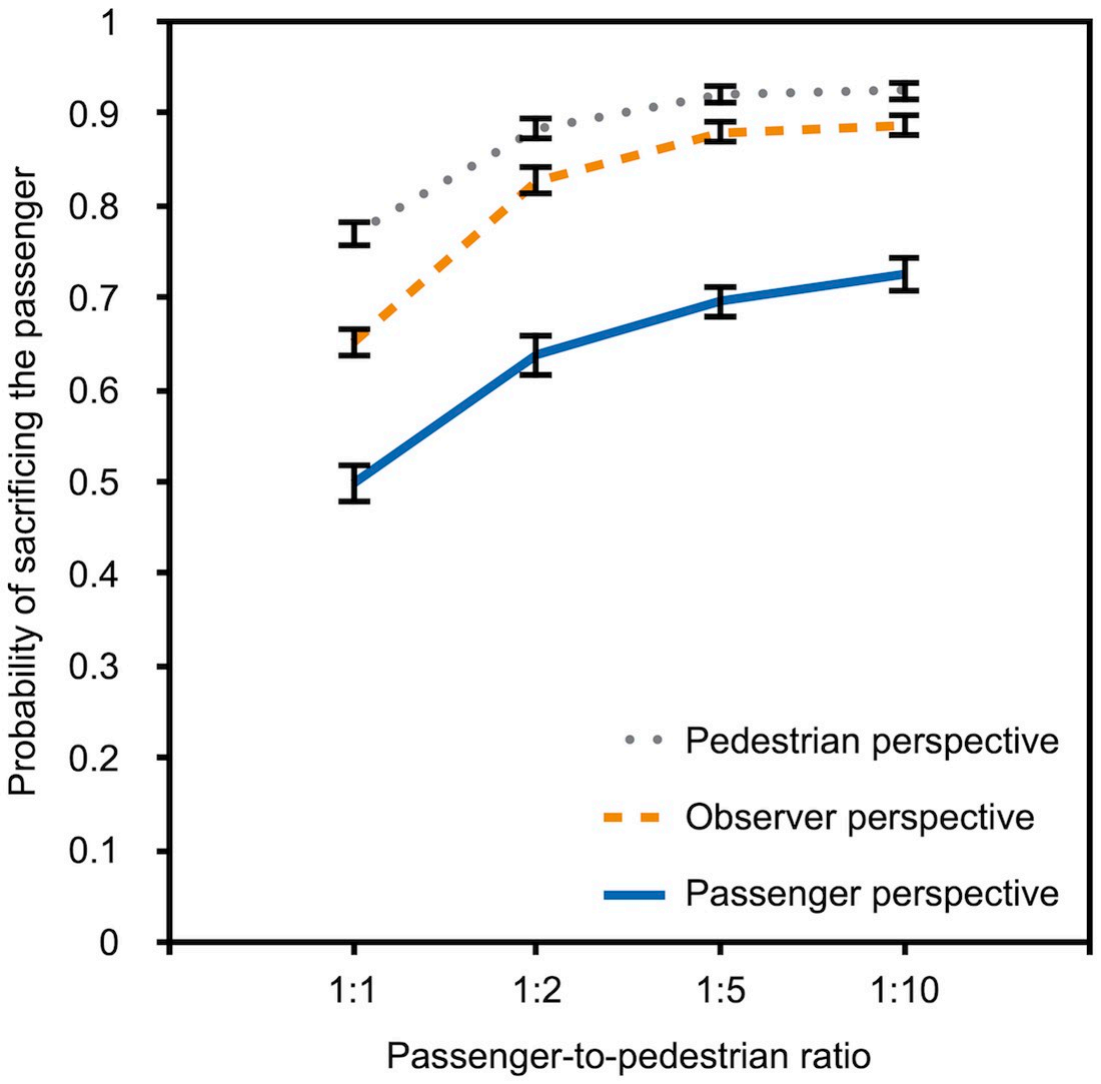
Descriptive data for Experiment 1b. The probability of sacrificing the passenger rather than the pedestrian/s is depicted as a function of passenger-to-pedestrian ratio (1:1, 1:2, 1:5, and 1:10) and perspective (pedestrian, observer, and passenger). The error bars represent bootstrapped standard errors.

The results were analyzed as in Experiment 1a. The participants’ preferences are shown in Fig 4. Due to technical difficulties with the display of one scenario, three (instead of four) responses were analyzed for the passenger-to-pedestrian ratio of 1:5.

The results displayed in Fig 4 suggest that the preference for sacrificing the passenger increases with an increasing number of pedestrians that can be saved by this action. The results also suggest that the preference to sacrifice the passenger differs as a function of perspective. Participants who had adopted the perspective of a pedestrian showed the strongest preference for sacrificing the passenger while participants who had adopted the perspective of a passenger showed the lowest preference for sacrificing the passenger at all levels of the passenger-to-pedestrian ratio variable. Confirming the visual impression from Fig 4, the multinomial analysis confirmed that the preferences of pedestrians differed significantly from those of passengers, *G*^*2*^(4) = 292.34, *p* < .001, *w* = .23. The preferences of pedestrians, *G*^*2*^(4) = 30.58, *p* < .001, *w* = .07, and passengers, *G*^2^(4) = 149.58, *p* < .001, *w* = .17, differed from those of observers.

Next, we compared the preferences for sacrificing the passenger among the perspectives at each level of the passenger-to-pedestrian ratio variable (Table 2). All pairwise comparisons are significant with the exception of the comparisons between pedestrians and observers at the passenger-to-pedestrian ratios of 1:5 and 1:10, which is probably due to the fact that an overwhelming majority of the pedestrians and observers preferred the utilitarian option of sacrificing the passenger in order to save the lives of five or more pedestrians. The passengers’ preferences differed from those of pedestrians and observers even at these extreme passenger-to-pedestrian ratios. These findings replicate those obtained in Experiment 1a.

**Table 2:** Comparisons among perspectives separately for each passenger-to-pedestrian ratio in Experiment 1b.

	1:1	1:2	1:5	1:10
Pedestrian vs. passenger	*G*^2^(1) = 77.55	*G*^2^(1) = 82.49	*G*^2^(1) = 61.84	*G*^2^(1) = 70.46
	*p* < .001*	*p* < .001*	*p* < .001*	*p* < .001*
	*w* = .12	*w* = .12	*w* = .11	*w* = .11
Pedestrian vs. observer	*G*^2^(1) = 16.39	*G*^2^(1) = 6.34	*G*^2^(1) = 3.55	*G*^2^(1) = 4.31
	*p* < .001*	*p* = .012*	*p* = .060	*p* = .038
	*w* = .05	*w* = .03	*w* = .03	*w* = .03
Observer vs. passenger	*G*^2^(1) = 23.84	*G*^2^(1) = 45.49	*G*^2^(1) = 37.93	*G*^2^(1) = 42.32
	*p* < .001*	*p* < .001*	*p* < .001*	*p* < .001*
	*w* = .07	*w* = .09	*w* = .08	*w* = .09

The α level was set to .05 and Bonferroni-Holm adjusted [54]. Significant comparisons are indicated by an asterisk.

### Discussion

The results of Experiment 1b replicate the main findings of Experiment 1a, suggesting that these findings are robust. Most importantly, the participants’ preferences for the action of autonomous vehicles in moral dilemmas is determined by their perspective. Participants who evaluated the scenarios from the perspective of a pedestrian had a stronger preference for sacrificing the passenger to save the pedestrian/s than participants who were cued into the perspective of the passenger. Even though differences between the pedestrian perspective and the passenger perspective were obtained at the most extreme passenger-to-pedestrian ratios, the results hint at the possibility that some degree of consensus can be reached about the preferred action of the autonomous vehicle as a majority of the participants agreed that the passenger should be sacrificed to save the lives of two or more pedestrians. Even a majority of the participants who were cued into the perspective of a passenger showed this utilitarian preference to save a maximum number of lives.

However, based on the results of Experiments 1a and 1b, we do not yet know whether pedestrians show a complementary preference for sacrificing a pedestrian to save the lives of several passengers of an autonomous vehicle. It is worth repeating here that the Ethics Commission of the German Federal Ministry of Transport and Digital Infrastructure [39] has granted a special status to road users outside of autonomous vehicles as they have argued that those who do not generate a mobility risk such as pedestrians must never be sacrificed to save those generating that risk such as passengers of an autonomous vehicle. If laypeople share the same moral believes, it is not certain that participants who are cued into the perspective of pedestrians will show an increasing preference for sacrificing a pedestrian to save the lives of two or more passengers of an autonomous vehicle. Instead, they may show a persistent preference to spare the pedestrian regardless of the number of passengers that could be saved by taking a different course of action. To test this hypothesis, we manipulated the number of passengers who could be saved by sacrificing a pedestrian in Experiments 2a and 2b.

## Experiment 2a

### Method

#### Participants

Participants were recruited and compensated as in Experiment 1a. Only participants who did not participate in Experiment 1a were allowed to participate. Of the participants who started the study, 50 did not complete the experiment, six were not of legal age, and 34 were excluded because they did not respond to all items. The final sample included the data of 312 participants (232 female, 80 male), aged between 18 and 63 years (*M* = 25, *SD* = 8). With this sample size and 16 evaluations, effects of size *w* = .06 could be detected at an α level of .05 with a statistical power of 1—β = .95 in the overall comparison among perspectives (*df* = 4). As in the previous experiments, participants were randomly assigned to one of three perspectives—pedestrian (*n* = 103), observer (*n* = 107), or passenger (*n* = 102)—from which they were asked to evaluate the moral dilemma scenarios. Additional information about the sample is reported at the OSF project page (https://osf.io/4xhz7/).

#### Material and procedure

Material and procedure were identical to those of Experiment 1a with the following exception. Instead of varying the number of pedestrians on the road, we now manipulated the number of passengers of the autonomous vehicle (within-subjects factor). Accordingly, there were one, two, five, or ten passengers inside the vehicle, but there was only one pedestrian. Thus, the experiment employed a 3 (perspective: pedestrian, observer, passenger; between-subjects factor) × 4 (passenger-to-pedestrian ratio: 1:1, 2:1, 5:1, 10:1; within-subjects factor) design.

### Results

**Fig 5:**
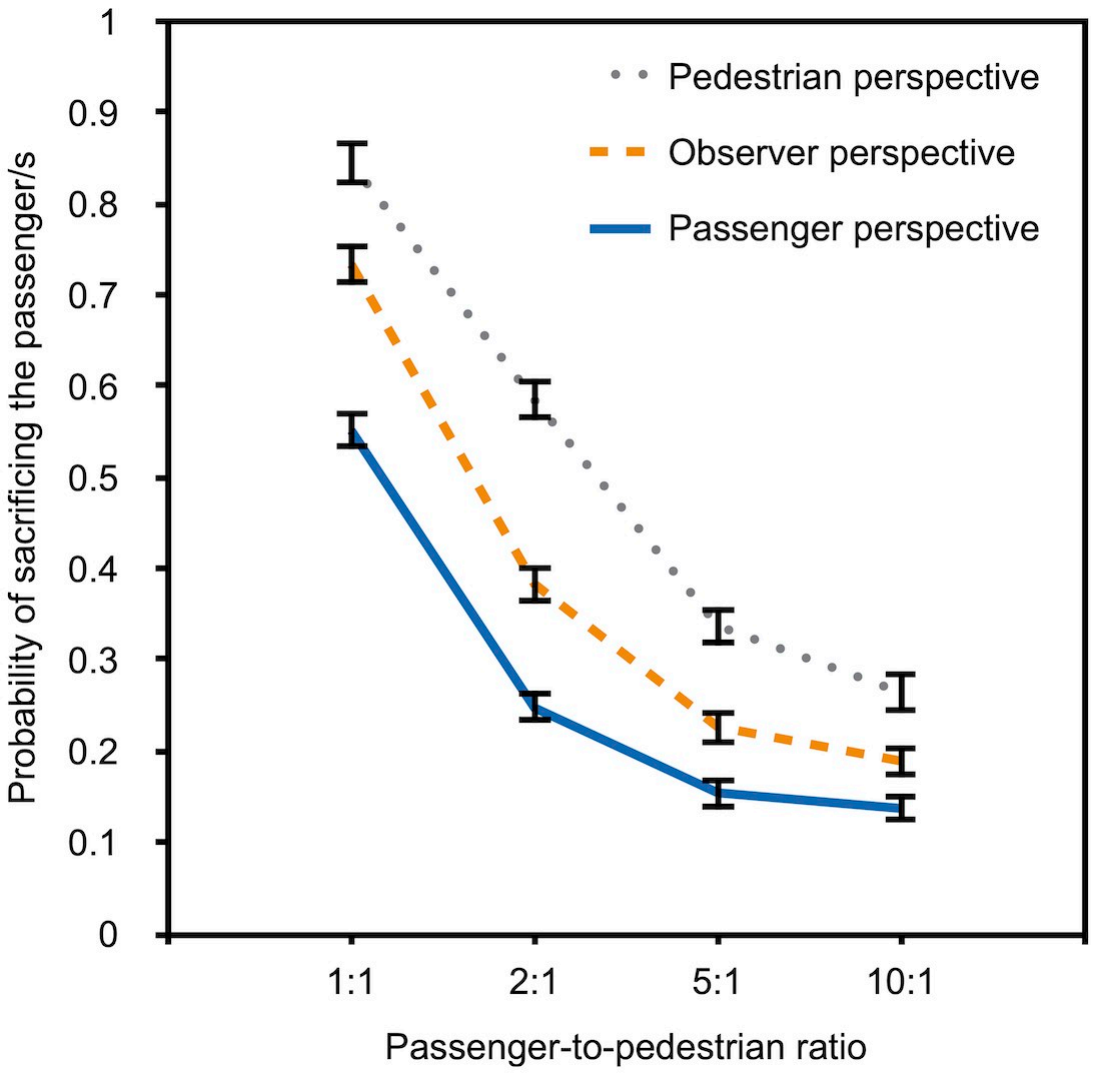
Descriptive data for Experiment 2a. The probability of sacrificing the passenger/s rather than the pedestrian is depicted as a function of passenger-to-pedestrian ratio (1:1, 2:1, 5:1, and 10:1) and perspective (pedestrian, observer, and passenger). The error bars represent bootstrapped standard errors.

Results were analyzed in the same way as in the previous experiments. The participants’ preferences are shown in Fig 5.

The first thing that seems noticeable is that, just as in the previous experiments, most participants prefer to sacrifice the passenger rather than the pedestrian when the passenger-to-pedestrian ratio is 1:1. However, there is an increasingly strong preference reversal with an increasing number of passengers whose lives can be saved by crashing into the pedestrian. As in the previous experiments, there were strong self-protective biases in the participants’ preferences. Participants who had adopted the perspective of a pedestrian showed the strongest preference for sacrificing the passenger while participants who had adopted the perspective of the passenger showed the lowest preference for sacrificing the passenger. Confirming the visual impression from Fig 5, the multinomial analysis confirmed that the preferences of pedestrians differed significantly from those of the passengers, *G*^2^(4) = 243.30, *p* < .001, *w* = .22. The preferences of pedestrians, *G*^2^(4) = 69.74, *p* < .001, *w* = .12, and passengers, *G*^2^(4) = 59.58, *p* < .001, *w* = .11, differed from those of observers.

Next, we compared the preferences for sacrificing the passenger among the perspectives at each level of the passenger-to-pedestrian ratio variable (Table 3). All pairwise comparisons are significant.

**Table 3:** Comparisons among perspectives separately for each passenger-to-pedestrian ratio in Experiment 2a.

	1:1	2:1	5:1	10:1
Pedestrian vs. passenger	*G*^2^(1) = 86.38	*G*^2^(1) = 98.24	*G*^2^(1) = 37.70	*G*^2^(1) = 20.98
	*p* < .001*	*p* < .001*	*p* < .001*	*p* < .001*
	*w* = .13	*w* = .14	*w* = .09	*w* = .06
Pedestrian vs. observer	*G*^2^(1) = 15.68	*G*^2^(1) = 34.46	*G*^2^(1) = 12.79	*G*^2^(1) = 6.82
	*p* < .001*	*p* < .001*	*p* < .001*	*p* = .009*
	*w* = .06	*w* = .08	*w* = .05	*w* = .04
Observer vs. passenger	*G*^2^(1) = 30.46	*G*^2^(1) = 17.88	*G*^2^(1) = 7.09	*G*^2^(1) = 4.14
	*p* < .001*	*p <* .001*	*p* = .008*	*p* = .042*
	*w* = .08	*w* = .06	*w* = .04	*w* = .03

The α level was set to .05 and Bonferroni-Holm adjusted [54]. Significant comparisons are indicated by an asterisk.

### Discussion

As in the previous experiments, most participants preferred to sacrifice the passenger rather than the pedestrian when the life of a passenger had to be weighed against the life of a pedestrian (passenger-to-pedestrian ratio 1:1). This mirrors the conviction expressed by the official guidelines of the Ethics Commission of the German Federal Ministry of Transport and Digital Infrastructure [39] that the life of a pedestrian should not be sacrificed to save the passenger of the autonomous vehicle. However, laypeople’s moral intuition assessed in Experiment 2a are much less rigid than the recommendations of the Ethics Commission. With an increasing number of passengers whose lives can be saved by sacrificing the pedestrian, preferences shift towards sacrificing the pedestrian. Even those participants who were cued into the perspective of a pedestrian show a utilitarian preference for sacrificing the pedestrian to save that of several passengers. Nevertheless, the results also confirm that differences among the perspectives are not completely eliminated even at the most extreme passenger-to-pedestrian ratios, showing a strong influence of self-protective biases on moral decision making. Again, we thought it desirable to test the robustness of these findings by performing a close replication with participants who were recruited from an online research panel.

## Experiment 2b

### Method

#### Participants

Participants were recruited and compensated as in Experiment 1b. None of the participants had participated in Experiment 1b. Of the participants who started the study, 36 did not complete the experiment, two were excluded because they indicated that they had insufficient German language skills to understand the instructions, 43 did not respond to all items, and 10 gave identical answers to all items of the three questionnaires. The final sample included the data of 388 participants (180 female, 208 male), aged between 19 and 69 years (*M* = 48, *SD* = 13). With this sample size and 16 evaluations, effects of size *w* = .05 could be detected at an α level of .05 with a statistical power of 1—β = .95 in the overall comparison among perspectives (*df* = 4). As in the previous experiments, participants were randomly assigned to one of three perspectives—pedestrian (*n* = 133), observer (*n* = 123), or passenger (*n* = 132)—from which they were asked to evaluate the moral dilemma scenarios. Additional information about the sample is reported at the OSF project page (https://osf.io/4xhz7/).

#### Material and procedure

Material and procedure were identical to those of Experiment 2a.

### Results

**Fig 6:**
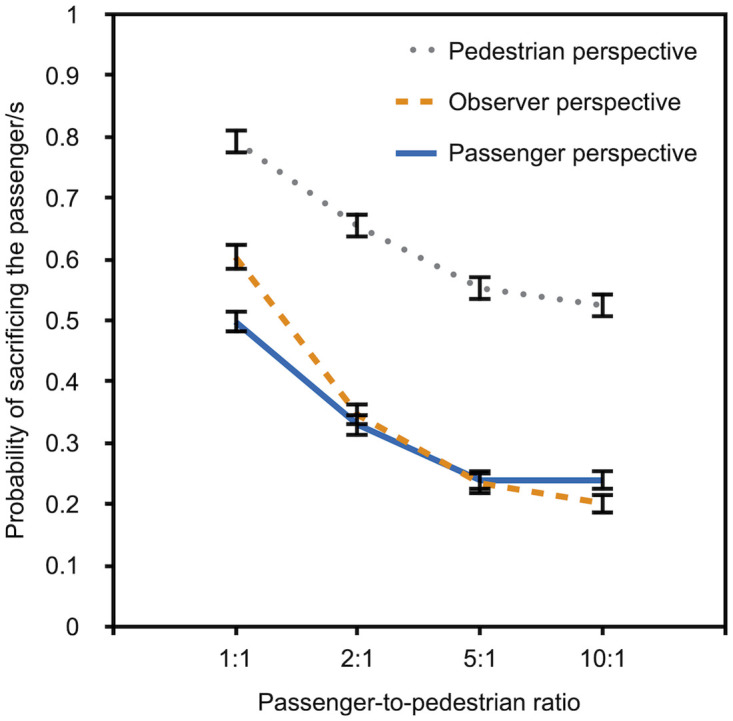
Descriptive data for Experiment 2b. The probability of sacrificing the passenger/s rather than the pedestrian is depicted as a function of passenger-to-pedestrian ratio (1:1, 2:1, 5:1, and 10:1) and perspective (pedestrian, observer, and passenger). The error bars represent bootstrapped standard errors.

The results were analyzed in the same way as in the previous experiments. The participants’ preferences are shown in Fig 6.

The results show that the majority of the participants (with the exception of those who were cued into the perspective of a passenger) had a preference for sacrificing the passenger when the passenger-to-pedestrian ratio is 1:1. However, the preference for sacrificing the passenger/s decreases with an increasing number of passengers whose lives can be saved by crashing into the pedestrian. Just as in the previous experiments, the preference to sacrifice the passenger differed as a function of perspective. Participants who had adopted the perspective of the pedestrian showed a much stronger preference for sacrificing the passenger to save the pedestrians than those who were cued into the perspective of a passenger, *G*^2^(4) = 422.06, *p* < .001, *w* = .26. The preferences of pedestrians, *G*^2^(4) = 372.76, *p* < .001, *w* = .25, and passengers, *G*^2^(4) = 13.89, *p* = .008, *w* = .05, differed from that of observers.

Next, we compared the preferences for sacrificing the passenger among the perspectives at each level of the passenger-to-pedestrian ratio (Table 4). All pairwise comparisons are significant with the exception of the comparisons between observers and passengers at the passenger-to-pedestrian ratios of 2:1, 5:1, and 10:1. This could be attributed to the utilitarian preference of observers to minimize harm and save a maximum number of lives.

**Table 4:** Comparisons among perspectives separately for each passenger-to-pedestrian ratio in Experiment 2b.

	1:1	2:1	5:1	10:1
Pedestrian vs. passenger	*G*^2^(1) = 103.26	*G*^2^(1) = 113.76	*G*^2^(1) = 111.60	*G*^2^(1) = 93.44
	*p* < .001*	*p* < .001*	*p* < .001*	*p* < .001*
	*w* = .13	*w* = .14	*w* = .13	*w* = .12
Pedestrian vs. observer	*G*^2^(1) = 44.28	*G*^2^(1) = 98.99	*G*^2^(1) = 111.18	*G*^2^(1) = 118.31
	*p* < .001*	*p* < .001*	*p* < .001*	*p* < .001*
	*w* = .08	*w* = .13	*w* = .13	*w* = .14
Observer vs. passenger	*G*^2^(1) = 11.49	*G*^2^(1) = 0.29	*G*^2^(1) = 0.03	*G*^2^(1) = 2.08
	*p* = .001*	*p* = .590	*p* = .854	*p* = .149
	*w* = .04	*w* = .01	*w* < .01	*w* = .02

The α level was set to .05 and Bonferroni-Holm adjusted [54]. Significant comparisons are indicated by an asterisk.

### Discussion

Overall, there is a high degree of consistency across all four experiments. Preferences about the action of an autonomous vehicle in a moral dilemma scenario strongly depends on perspective. Participants who evaluated the scenario from the perspective of the pedestrian consistently displayed the highest preference for sacrificing the passenger while participants who were cued into the perspective of the passenger showed the lowest preference for sacrificing the passenger. This suggests that these preferences are strongly affected by self-protective biases. However, there is some degree of agreement among all of the perspectives. With an increasing number of lives that can be saved through sacrificing either the passenger or the pedestrian, preferences for the utilitarian option of saving a greater number of lives increases. This implies that a considerable proportion of people are willing to self-sacrifice when a large number of people can be saved by such a selfless act.

However, a possible caveat is that these people may have chosen to self-sacrifice based on a social desirability bias [cf. 56]. That is, participants may openly indicate to favor the utilitarian option of sacrificing themselves to save the lives of many others because they want to avoid the embarrassment of being perceived as selfish by choosing the self-protective option. There is evidence that sacrificing someone else for one’s own good is seen as morally less acceptable than self-sacrificing [57]. It thus seems possible to speculate that some subset of participants may have chosen the self-sacrificing option only to present themselves in a favorable light. In other words, people may respond in line with what they perceive to be a moral norm instead of admitting to their self-protective preferences. If this were the case, then the results of Experiments 1a to 2b would have overestimated the preference for the socially desirable utilitarian option and underestimated the role of socially undesirable self-protective preferences.

Fortunately, there are questioning techniques that address the issue of social desirability. Indirect questioning techniques, such as the Randomized Response Technique [58], guarantee respondents confidentiality to counteract social desirability bias [for a more detailed introduction to indirect questioning techniques see e.g., 59]. The underlying idea is to add obvious random noise to the data so that it is not possible to determine, at an individual level, what answer the respondent gave to the sensitive question which assesses the attribute potentially affected by social desirability. In consequence, the influence of social desirability on responding is reduced and the corresponding prevalence estimates are considered more valid compared to conventional direct questioning approaches [60]. To illustrate, participants may be presented with the statements “I have never driven under the influence of alcohol” and “I have driven under the influence of alcohol”. Unobserved by the experimenter, they then roll a dice to determine whether they respond to the first or the second statement with “yes” or “no”. Given that the interviewer does not know to which statement the answer belongs, one can assume that participants are more willing to answer truthfully. However, provided that the randomization probability is known, the prevalence of the sensitive attribute can be determined at the group level. Since the Randomized Response Technique has been proposed, indirect questioning techniques have been improved to address limitations of the original method such as relying on an external randomization device. The Crosswise Model [61] requires participants answer two questions or evaluate two statements at once. One of these refers to the sensitive attribute (e.g., “I have driven under the influence of alcohol”) that is to be assessed while the other refers to an attribute with known prevalence. For example, the second statement may be “I was born in November or December”. The probability of a “yes” response to the second statement can be estimated from official birth statistics. Participants then have to choose between the options “I agree with both statements or with neither statement” and “I agree with only one of the statements (irrespective of which one)”. The Crosswise Model is mathematically identical to the Randomized Response Technique but it has the advantage that it does not require an external randomization device (as the non-sensitive statement is used for adding random noise to the data). Another advantage of this procedure is that it does not offer participants a “safe” response option such as “no”. It is also easier to understand than other indirect questioning techniques [62]. In line with the assumption that the increased confidentiality of responding reduces the influence of socially desirability, the Crosswise Model leads to higher estimates of socially undesirable attitudes, preferences, and behaviors such as tax evasion [63], plagiarism in student papers [64], distrust [65], prejudice against women leaders [66], xenophobia, and islamophobia [67]. What is more, the Crosswise Model leads to more accurate estimates of cheating behavior whose prevalence is known [68]. In the present study, we will rely on the Extended Crosswise Model [45]. This extension of the Crosswise Model [61] has the additional advantage that one can detect whether participants systematically deviate from the instructions (e.g., by misunderstanding the instructions or by responding carelessly) and thus allows to test the validity of the data without a loss in efficiency. This model has been successfully validated [45] and was favorably evaluated in a recent experimental application [69].

If the participants’ answers in response to moral dilemmas with autonomous vehicles that involve self- and other-sacrifices were biased by socially desirable responding, the indirect questioning approach should yield higher approval for the sacrificing of several other people to save one’s own life than the direct questioning approach. In consequence, the approval for the socially desirable option to self-sacrifice should decrease. To illustrate, in a study conducted in an early phase of the COVID-19 pandemic on the compliance with the precautionary measures against infections with the SARS-CoV-2 virus [70], 94.5% of the participants claimed to wash their hands regularly and sufficiently long with soap and water in response to a direct question but the indirect questioning approach yielded a significantly smaller prevalence estimate of 78.1%. By comparing estimates that are based on the Extended Crosswise Model [45] and a direct question, it is possible to test whether, and to what degree, direct self-reports are contaminated by social desirability. To simplify the analysis, participants were asked to evaluate only one scenario in Experiment 3. A passenger-to-pedestrian ratio of 5:1 was selected because previous evidence suggests that a group size of five represents a switching point. In a study of Faulhaber et al. [18], the participants’ willingness to self-sacrifice in order to save others increased when the number of lives that could be saved by the selfless act increased from one to five but it did not increase further beyond this point. The data of Experiments 1a to 2b reported here also indicate only small changes in the willingness to sacrifice the passenger between a group of five and a group of 10 (cf. Figs 3–6). There thus seems to be a comparatively strong utilitarian norm to self-sacrifice in order to save five other lives. If the preference for this utilitarian norm to save the lives of others is partly or fully caused by social desirability bias, the preference to self-sacrifice should be decreased in the indirect questioning condition in comparison to the direct self-reports.

## Experiment 3

### Method

#### Participants

Participants were recruited and compensated as in Experiments 1b and 2b. Of the participants who started the study, 79 did not complete the experiment, 13 were not of legal age, indicated that they were unable to properly read the text presented on the screen or that they had insufficient German language skills, and another two data sets were excluded because of double participation. The final sample consisted of *N* = 1,380 participants (621 female, 756 male, 3 diverse) aged between 18 and 99 years (*M* = 55, *SD* = 13). With this sample size, effects of *w* = .10 [that is, a small effect according to 48] could be detected at an α level of .05 with a statistical power of 1—β = .95 in the comparison of the preference estimates between the direct questioning approach and the indirect questioning approach (*df* = 1). Participants were randomly assigned to one of three experimental groups (between-subjects factor; see explanation below): Direct Questioning Group (*n* = 459), Indirect Questioning Group 1 (*n* = 461), and Indirect Questioning Group 2 (*n* = 460).

#### Materials and procedure

**Fig 7:**
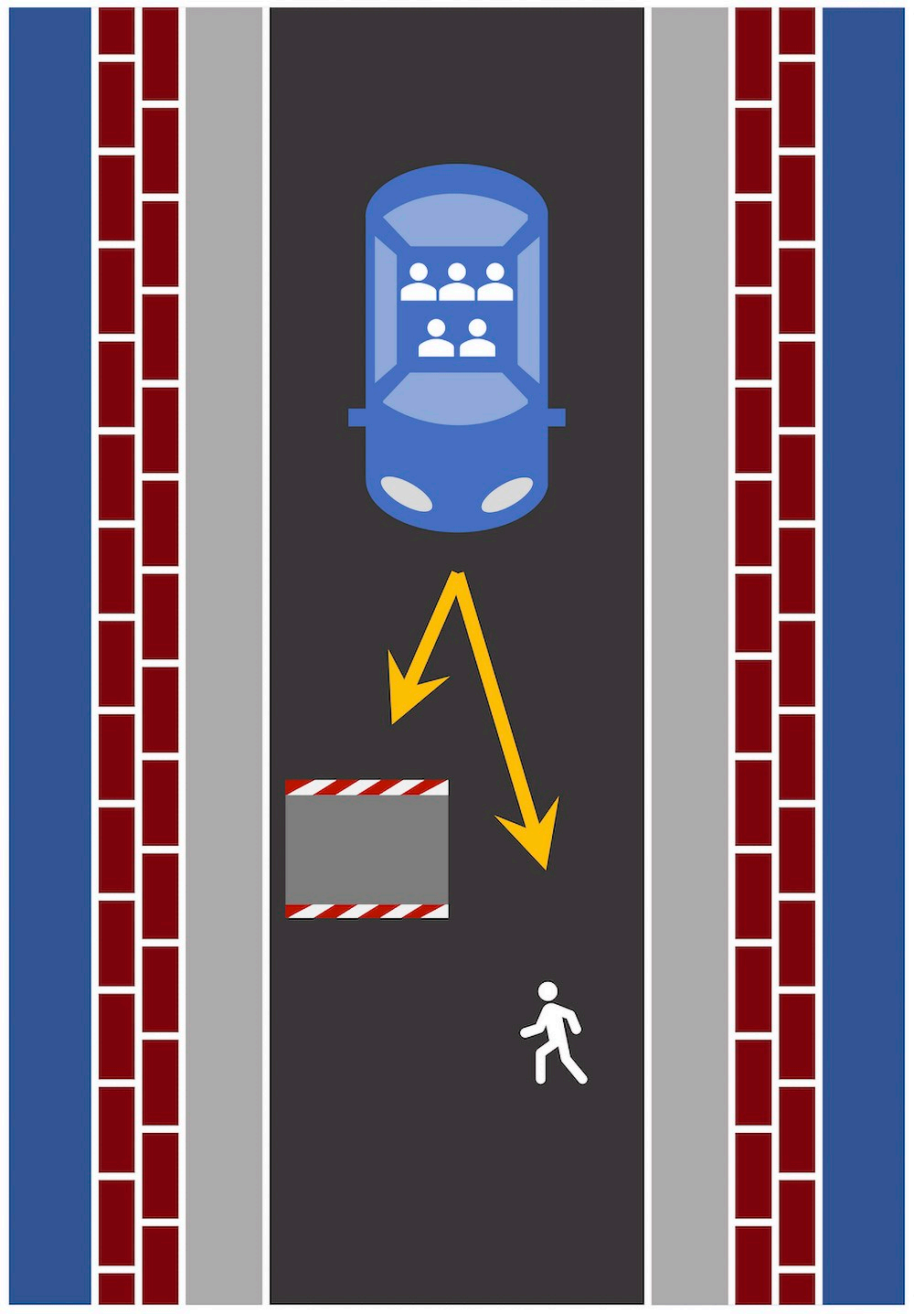
The illustration that was used for the moral dilemma scenario. The passenger-to-pedestrian ratio was 5:1, which implies that the life of five passengers was weighed against that of one pedestrian. The visual illustration of the scenario were created using Microsoft PowerPoint.

All participants were asked to adopt the perspective of the pedestrian. They saw only one scenario in which the life of one pedestrian had to be weighed against the lives of five passengers inside the autonomous vehicle. The image showed an autonomous vehicle on a single-lane road heading towards a roadblock and a single pedestrian on the road from a bird’s eye view (Fig 7). In a written account of the incident, participants were informed that the accident would inevitably result in the death of either the passengers or the pedestrian.

Below the image and the vignette, participants were asked to evaluate the depicted scenario from the perspective of the pedestrian. Participants in the Direct Questioning Group were asked to evaluate the sensitive statement “In the depicted situation, the autonomous vehicle should sacrifice the five passengers to save me as a pedestrian” with “True” or “False”.

Participants in the indirect questioning groups received instructions on the indirect questioning technique explaining to them that it was impossible to infer, at an individual level, their true opinion on the matter from the answers that were given. As in previous studies [e.g., 65, 66, 71], the month of birth was used as the non-sensitive attribute in the two indirect questioning groups. Participants in the indirect questioning groups also knew that we did not know their month of birth and that they would not be asked about it. Accordingly, when presented with the scenario following the instructions, participants in Indirect Questioning Group 1 were asked to evaluate the sensitive statement together with the non-sensitive statement “I was born in November or December” by choosing from the answer categories “I either agree with both statements or with neither statement” and “I agree with only one statement (irrespective of which one)”. Participants in Indirect Questioning Group 2 were provided with the same answer categories, but the non-sensitive statement was replaced by the complementary non-sensitive statement “I was born between January and October”.

The experiment thus employed a group design with three experimental groups (Direct Questioning Group, Indirect Questioning Groups 1 and 2). In total, participation in the experiment took about 5 minutes.

### Results

**Fig 8:**
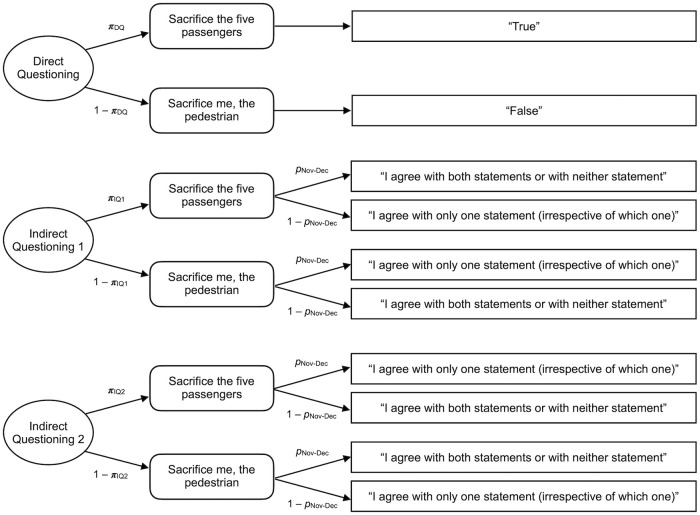
Multinomial processing tree model. The combined multinomial processing tree model for the Direct Questioning Group—represented by the upper tree—and for Indirect Questioning Groups 1 and 2—represented by the lower two trees—for the Extended Crosswise Model [45] adapted to the present experiment. The rectangles on the right contain the answer categories available in each condition. Parameter π represents the prevalence estimates for the preferences that the autonomous vehicle should sacrifice five passengers of the autonomous vehicle in order to save the pedestrian, depending on the condition. Parameter *p*_Nov-Dec_ represents the known prevalence of the non-sensitive attribute, in this case, the participant being born in November or December.

As in the previous experiments, we used *multiTree* [53] to estimate the preference for sacrificing the passengers based on the observed answer frequencies and to compare these preferences among the groups. The Extended Crosswise Model [45] as used here is shown in Fig 8.

In the Direct Questioning Group (see upper tree in Fig 8), the prevalence π_DQ_ of the sensitive attribute (preference for sacrificing the five passengers to save the pedestrian) corresponds directly to the probability that the answer category “True” was obtained. Note that the upper tree corresponds to the way in which the parameters were obtained in the previous experiments (Fig 2). Obtaining the prevalence estimates for the sensitive attribute in the indirect questioning groups (lower two trees in Fig 8) is somewhat more complex as participants’ true status on the assessed attributes cannot be directly inferred from the provided answers. Parameters π_IQ1_ and π_IQ2_ represent prevalence estimates of the sensitive attribute. Parameter *p*_Nov-Dec_ represents the known prevalence of being born in November or December. The respective prevalence can be derived from official birth statistics. According to the German birth statistics, the probability of being born in November or December is approximately 15.8% [72]. Hence, we set parameter *p*_Nov-Dec_ to .158 in the following analyses. To obtain a prevalence estimate of the month of birth in the present sample, participants in the Direct Questioning Group were asked to indicate whether they were born between January and October. The statistical conclusions do not change when the prevalence estimate is based on the sample prevalence estimate of *p*_Nov-Dec_ *=* .176. Participants in the two indirect questioning groups evaluated the sensitive and the non-sensitive statement simultaneously. The only difference between the two indirect questioning groups was the non-sensitive statement. In Indirect Questioning Group 1 the non-sensitive statement was “I was born in November or December” (*p*_Nov-Dec_), in the Indirect Questioning Group 2 it was “I was born between January and October” (1 –*p*_Nov-Dec_). The answer categories depicted in Fig 8 are therefore swapped for Indirect Questioning Group 2 (“Indirect Questioning 2” in Fig 8) in comparison to Indirect Questioning Group 1 (“Indirect Questioning 1” in Fig 8).

As noted earlier, the Extended Crosswise Model allows to test whether participants follow the instructions. Specifically, the prevalence estimates for the sensitive attribute must not differ between the two indirect questioning groups when participants follow the instructions. An analysis of the Extended Crosswise Model thus starts with equating the prevalence estimates of the two indirect questioning groups. All subsequent model-based results can only be trusted when it is possible to combine the two parameters π_IQ1_ and π_IQ2_ into a single parameter π_IQ_ representing the prevalence estimate based on indirect questioning. The model assuming that the prevalence estimates do not differ between the two groups (π_IQ1_ = π_IQ2_) fitted the data well, *G*^*2*^(1) = 1.37, *p* = .243, *w* = .03. According to Heck et al. [45], this indicates that participants adhered to the instructions and “the prevalence estimate can be considered trustworthy” (p. 1897). Therefore, the two indirect questioning groups were pooled for further analysis.

Next, we tested whether the prevalence estimates differed between the direct questioning approach and the indirect questioning approach. In the Direct Questioning Group (*n* = 459), 41.0% (*SE* = 2.3) indicated that the autonomous vehicle should sacrifice five passengers to save them while the prevalence estimate for the sensitive attribute in the Combined Indirect Questioning Group (*n* = 921) was 40.1% (*SE* = 2.4). The assumption that the prevalence estimates did not differ between the Direct Questioning Group and the Combined Indirect Questioning Group (π_DQ_ = π_IQ_) was compatible with the data, Δ*G*^*2*^(1) = 0.07, *p* = .790, *w* = .01. This indicates that the prevalence estimates did not differ between the direct and the indirect questioning approach. In other words, the hypothesis that the prevalence estimates based on the direct questioning approach are compromised by social desirability must be rejected.

### Discussion

Experiment 3 served to test whether direct self-reports of a (utilitarian) self-sacrificing preference in a moral dilemma with autonomous vehicles are compromised by social desirability. To this end, we used the Extended Crosswise Model [45] to test whether increased confidentiality of responding would decrease the approval of the self-sacrificing option. Disconfirming the hypothesis that the utilitarian preference for self-sacrifice is only due to social desirability, preference estimates did not differ between the direct and the indirect questioning. Participants expressed the preference to sacrifice themselves to save the lives of five others even when a high degree of confidentiality was guaranteed. This is all the more interesting given that the indirect questioning technique used here has been shown to reliably reveal effects of social desirability on answers to questions about sensitive topics such as prejudice against Muslims and hand hygiene [69, 70].

This indicates that people’s preference for the utilitarian option of sacrificing themselves to save the lives of five other people was not, or at least not to an appreciable degree, affected by social desirability [see 33, for further evidence that the influence of social desirability on people’s preferences in moral dilemmas is limited]. It also seems noticeable that the results of Experiment 3 are well aligned with the results of the previous experiments. There is an overall preference for the autonomous vehicle to save a maximum number of lives even if this means sacrificing oneself. Nevertheless, a substantial proportion of participants (about 40%) prefer the self-protective option even if this means to kill five other people.

## General discussion

Automated vehicle technologies promise benefits such as improved accessibility of transportation, and increased traffic safety [e.g., 1, 5]. Yet, before autonomous vehicles can be implemented on a large scale, several challenges need to be addressed—besides the technical implementation—for example issues regarding ensuring the safety of road users and passengers as well as software security, developing the legal requirements, and creating the necessary infrastructure [e.g., 2]. A hotly debated topic is how autonomous vehicles should handle accident situations [e.g., 10, 12–14, 18, 35, 73] and whether, and to what degree, people prefer actions of autonomous vehicles that are biased to save their own lives (as passengers or pedestrians) at the cost of those of others [e.g., 18, 35, 38]. These self-protective biases may clash with those of other road users, leading to potential conflicts that may slow or complicate the introduction of autonomous vehicles. To investigate to what extend preferences of non-motorized road users regarding moral dilemmas involving autonomous vehicles may differ from those of passengers, we compared the preferred action of an autonomous vehicle from the perspectives of a passenger, a pedestrian, and an observer in moral dilemma scenarios involving a varying number of potential victims. Our results suggest that perspective strongly determines the preferred course of action. Specifically, people cued into the perspective of passengers consistently expressed the least preference for sacrificing the passenger/s of the autonomous vehicle while pedestrians consistently expressed the highest preference for sacrificing the passenger/s.

Scenarios commonly employed to investigate moral dilemmas with autonomous vehicles often feature passengers and pedestrians [e.g., 18, 35, 40, 73] but, as yet, only few studies [43, 44] have required participants to evaluate the scenarios from the perspective of the pedestrians. The results strongly indicate that evaluations from the pedestrian perspective are important as pedestrians and passengers evaluate moral dilemmas with autonomous vehicles very differently. This is because the pedestrians, just like the passengers, display clear self-protective tendencies.

Given that passengers and pedestrians differ in their preferences for how autonomous vehicles should handle accident situations, the question arises of how the conflicting positions can be reconciled. Even though the present results show pervasive self-protective biases across all experiments, the results also suggest that it might be possible to reach some degree of agreement among the perspectives. A majority of those participants who were cued into the perspective of an uninvolved observer preferred protecting the pedestrian when the passenger-to-pedestrian ratio was 1:1 but preferred the utilitarian option of sparing maximum lives in all other conditions. With an increasing number of lives at stake, more and more participants preferred the utilitarian option of sparing a maximum number of lives even at the cost of sacrificing their own lives. This seems to imply that not all people want to save their own life at all cost. This was true both for passengers (Experiments 1a and 1b) and pedestrians (Experiments 2a and 2b). This suggests that, contrary to official guidelines [39], pedestrians may be willing to accept some degree of risk caused by autonomous vehicles.

At first sight it seemed possible to assume that this self-sacrificing tendency could be attributed to social desirability bias. However, this hypothesis has to be rejected given the results of Experiment 3. Even when an indirect questioning technique [45] guaranteed confidentiality of responding, the majority of the participants (about 60%) expressed the preference for sacrificing themselves to save the five passengers inside the autonomous vehicle and this majority was equally large when participants were questioned directly. The results suggest that the participants’ preference for a self-sacrifice to save the lives of several others is not only due to social desirability bias. Instead, it seems that they were privately convinced that the utilitarian option is the right course of action. This suggests that the preferences of passengers of autonomous vehicles and other road users can, to some degree, be reconciled with each other despite the persistent self-protective tendencies. More knowledge may be gained about how the differences between perspectives can be reconciled by examining the degree to which people’s preferences in moral dilemmas change depending on the degree to which it is emphasized that the same person might take different roles in traffic. This approach resembles the so-called veil-of-ignorance reasoning employed, for example, by Huang et al. [74]. In their study, participants were asked which option they would prefer in a moral dilemma if they did not know who among the affected parties they would be. Participants who engaged in this type of veil-of-ignorance reasoning displayed a higher preference for the utilitarian option in response to a subsequently presented dilemma than participants in a control condition. Thus, encouraging participants to consider that the role one takes in traffic varies may provide a means to reduce self-protective tendencies.

A limitation of the present research is that participants were asked to evaluate abstract scenarios. The decisions may thus be representative of situations such as when contemplating to purchase an autonomous vehicle in which people are able to make judgments about moral dilemmas without the imminent threat or stress of a real-life accident. It is unclear whether the preferences that were identified here generalize to decisions that are made in more extreme situations when life and death are a matter of seconds. Here it seems relevant that the present results are largely consistent with those of Kallioinen et al. [43] who manipulated the perspective from which an imminent accident was observed in an immersive virtual environment. Participants who experienced the scenario from a passenger perspective were less willing to put themselves at risk by guiding the autonomous vehicle off a cliff than participants who viewed the scene from the perspective of a pedestrian. It seems noticeable that the self-protection bias was limited in the study of Kallioinen et al. even though they used an immersive methodology in which the accident was experienced first-hand. In their first study, a conflict between the passenger and the pedestrian emerged only in a specific scenario in which serious harm to the passenger seemed likely. Possibly, scenarios that imply a clear self-sacrifice provide a higher potential for strong disagreement between the involved parties [25] than scenarios with more ambiguous consequences. Together, the results suggest that the self-protection bias is a pervasive cognitive bias that affects moral decision making both when being immersed in a critical traffic situation and when reasoning about abstract moral dilemmas.

Another limitation of the present study is that there is some culture-specific variation in moral preferences [13] so that it cannot be taken for granted that the findings reported here generalize across different samples. As a first step for testing the robustness of the present findings, we tested whether the results of Experiments 1a and 2a that were obtained with student samples (mostly young adults with little driving experience) could be replicated in Experiments 1b and 2b with samples from online research panels (adults with higher driving experience and more heterogeneous age and education). The fact that most of the results of the student samples could be replicated in the online samples is encouraging, as is the fact that the present results are largely consistent with those obtained in other labs in Denmark and Germany as well as international and US online samples [43, 44]. Nevertheless, most of the studies focused on well-educated Western samples so that examining the degree to which the self-protective and self-sacrificing preferences generalize to other samples is an interesting avenue for further research. Larger and more diverse samples than those used in the present study would be necessary to test how the self-protection bias is affected by potentially moderating factors such as gender, age, and personality.

In conclusion, the studies presented here aim at contributing to the discussion surrounding moral dilemmas involving autonomous vehicles. The perspective from which participants evaluated moral dilemma scenarios strongly affected the preferred action of the autonomous vehicle in the respective scenario. Specifically, passengers and pedestrians differed in their preferences from each other, but also from uninvolved observers, which suggests that self-protective biases have a strong influence on the evaluation of moral dilemmas involving autonomous vehicles. As a consequence of these conflicting interests, focusing on only one perspective may be problematic for the acceptance of autonomous vehicles in the long run. To guarantee widespread social acceptance, which is necessary for the success of autonomous vehicles [e.g., 13, 25, 32, 35], a careful balancing of the conflicting interests of the involved perspectives might be required. The present results suggest that some degree of consensus can be reached among the different perspectives. Regardless of the perspective, many participants preferred the utilitarian option of saving a maximum number of lives, even when the utilitarian option implied a self-sacrifice. Although differences among the perspectives did not completely vanish even when utilitarian principles clearly favored one of the available options, the majority of the participants who were cued into the perspective of the passenger agreed that the passenger should be sacrificed to save the lives of a group of pedestrians. Similarly, a majority of the participants who were cued into the role of the pedestrian agreed that the pedestrian should be sacrificed to save the lives of several passengers inside the autonomous vehicle. There is no evidence that the utilitarian preference for a self-sacrifice is caused by social desirability as participants expressed this preference even in an indirect questioning format that is known to reveal effects of social desirability. The results therefore suggest that, despite prevailing self-protective tendencies, there are some moral principles that all road users can agree upon.
